# Endoscopic Retrograde Cholangiopancreatography in the Diagnosis and Management of Choledochal Cysts

**DOI:** 10.1155/1997/38416

**Published:** 1997

**Authors:** Hikmet Akkiz, Salih O. Çolakoğlu, Yilmaz Ergün, Haluk Demiryürek, Alper Akinoğlu, Ïlhan Tuncer, Gürsel Özgür, Ali Hafta

**Affiliations:** 1 Departments of Gastroenterology University of Çukurova School of Medicine Adana Turkey; 2 Departments of Surgery University of Çukurova School of Medicine Adana Turkey; 3 Departments of Pathology University of Çukurova School of Medicine Adana Turkey

## Abstract

Choledochal cysts are an uncommon anomaly of the biliary system manifested by cystic dilatation of the extra or intrahepatic biliary tree or both. It is most frequently found in Orientals and in females. Endoscopic retrograde cholangiopancreatography is a valuable imaging technique in the diagnosis of choledochal cysts in adults. Additionaly, in selected cases, a choledochocele may be effectively managed by endoscopic sphincterotomy. We present clinical and endoscopic findings of six adult patients with choledochal cysts. Clinical symptoms were characterized by abdominal pain, jaundice and cholangitis. Associated hepatobiliary pathologic findings included cholelithiasis, recurrent acute pancreatitis, gallbladder carcinoma, Cystolithiasis, choledocholithiasis, biliary stricture and hepatic abscess.


*HPB Surgery*, 1997, Vol. 10, pp. 211–219
Reprints available directly from the publisher
Photocopying permitted by license only

© 1997 OPA (Overseas Publishers Association)
Amsterdam B.V. Published in The Netherlands
by Harwood Academic Publishers
Printed in India

### Endoscopic Retrograde Cholangiopancreatography in the Diagnosis and Management of Choledochal Cysts

HIKMET AKKIZ[a], SALIH O. ÇOLAKOĞLU[a], YILMAZ ERGÜN[a], HALUK DEMIRYÜREK[b], ALPER AKINOĞLU[b], İLHAN TUNCER[c], GÜRSEL ÖZGÜR and ALI HAFTA[a]

*Departments of [a]Gastroenterology, [b]Surgery and [c]Pathology, University of Çukurova School of Medicine, Adana, Turkey*

*(Received 26 June 1996)*

**Choledochal cysts are an uncommon anomaly of the biliary system manifested by cystic dilatation of the extra or intrahepatic biliary tree or both. It is most frequently found in Orientals and in females. Endoscopic retrograde cholangiopancreatography is a valuable imaging technique in the diagnosis of choledochal cysts in adults. Additionaly, in selected cases, a choledochocele may be effectively managed by endoscopic sphincterotomy. We present clinical and endoscopic findings of six adult patients with choledochal cysts. Clinical symptoms were characterized by abdominal pain, jaundice and cholangitis. Associated hepatobiliary pathologic findings included cholelithiasis, recurrent acute pancreatitis, gallbladder carcinoma, Cystolithiasis, choledocholithiasis, biliary stricture and hepatic abscess.**

*Keywords:* Choledochal cyst-Choledochocele-Surgery Endoscopy

### INTRODUCTION

Choledochal cysts are congenital anomalies of the biliary tract manifested by cystic dilatation of the extrahepatic and intrahepatic bile ducts [1,2]. Since Vater first described this entity in 1723, approximately 3000 cases have been reported worldwide [3–5]. Choledochal cysts are not familial and most frequently found in Orientals and in females [1,2,5]. More than two-thirds of the cases were reported from Japan with the incidence of 1/1,000 [2,5,6]. In western countries the estimated incidence is one in every 13,000 and 14,000 hospital admissions [2,6]. Although their origin is unknown, bile duct cysts are generally considered congenital [7]. Congenital weakness of the duct wall, a primary abnormality of epithelial proliferation during embryologic ductal development, and congenital obstruction have been suggested [2]. A high incidence (approximately 40 percent) of anomalous junction of the pancreatic and common bile ducts has been described that could potentially allow reflux of pancreatic enzymes into the bilary tree, leading to chronic inflammation, epithelial denudation, thinning of the bile duct wall and eventually cyst formation [2,8,9].

The classification of Todani *et al.* [10] of bile cysts is most often used, expanding on that Alonso-Lej *et al.* [7] by including intrahepatic cysts and further subdividing extrahepatic disease (Fig. 1). The classic triad pain, jaundice and abdominal mass occur in only a third of patients

Correspondence to: Hikmet Akkiz, M.D. Çukurova University Medical Faculty Department of Gastroenterology, Balcali, Adana, Turkey.

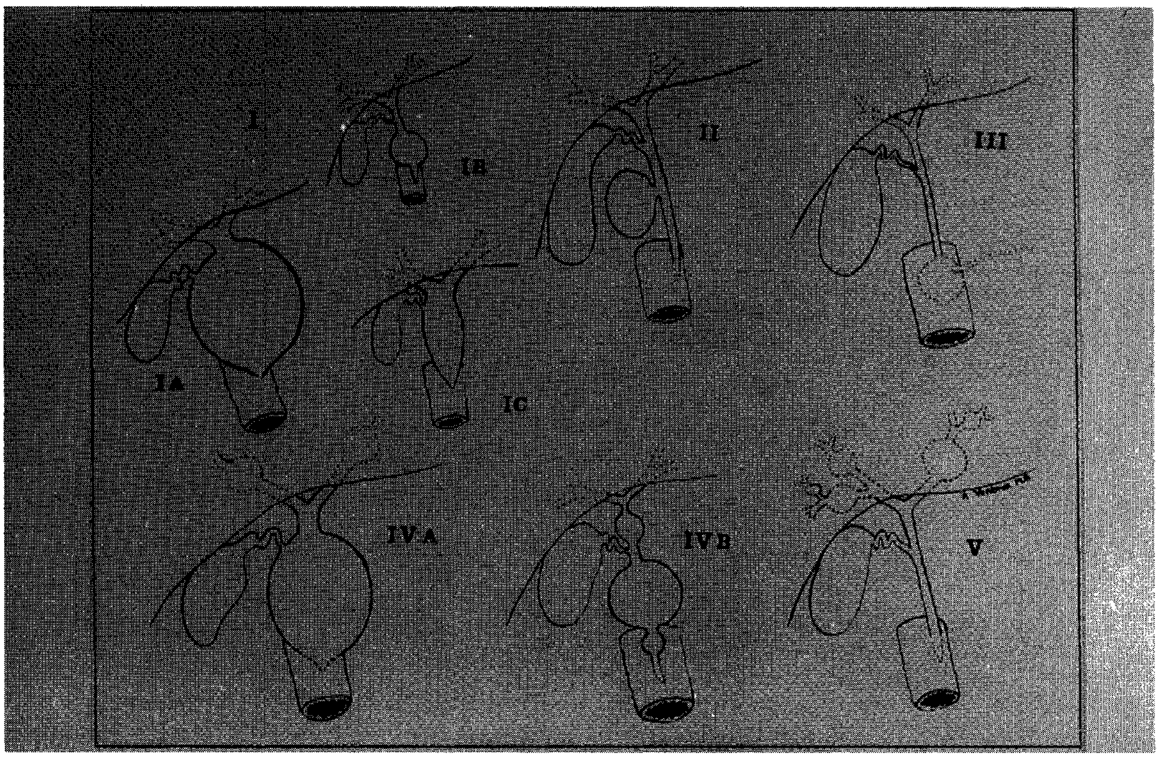

FIGURE 1 Classification of choledochal cysts according to Todani, *et al.* IA, common type; IB segmental dilatation; IC, diffuse dilatation; II, diverticulum; III, choledochocele; IVA, multiple cysts (intrahepatic and extrahepatic); IVB, multiple cysts (extrahepatic); V, single or multiple dilatations of the intrahepatic ducts.

[7,11]. Imaging technique such as ultrasound (US), computed tomography (CT), magnetic resonance imaging (MRI) are helpful in demonstrating a choledochal cyst [1,2,5,6,12,13]. Delination of the exact anatomy of the cyst and the intra-and extraheptic biliary tree is necessary to plan the operative treatment. In this regard, endoscopic retrograde cholangiopancreato graphy (ERCP) has been found to be useful in adults, but there are few reports of its use in pediatric patients [1,5,6,14–18]. The management of choledochal cysts are mainly surgical [1,2,5,14,17–23]. However in selected cases, a choledochocele may be treated by endoscopic sphincterotomy [14].

We would like to report clinical pictures, ERCP findings and hepatobiliary pathologies of six adult patients with choledochal cyst at our gastroenterology unit between January 1991 and December 1994.

### CASE REPORTS

**Case 1:** A 55-yr-old female was admitted to the hospital because of jaundice, fever and right upper quadrent (RUQ) pain. Two years before admission, she had undergone cholecystectomy because of cholelithiasis. Temperature, on admission, was 39.2°C. She was jaundiced and right upper abdomen was tender. Laboratory findings were as follows: Serum bilirubin, 129.2 µmol/l direct bilirubin 83.3 µmol/l; alkaline phosphatase, 675 U/L; leucocyte count, $16.4\times10^9$/l; γ-glutamyl transpeptidase (γ-GT), 455 U/L; Aspartate aminotransferase (AST), 102 U/L; Alanine aminotransferase (ALT), 86 U/L; serum amylase, 70 U/L; US and CT showed cystic dilatation of the common bile duct. ERCP findings were consistent with type II choledochal cyst (Fig. 2). At laparotomy, choledochal cyst

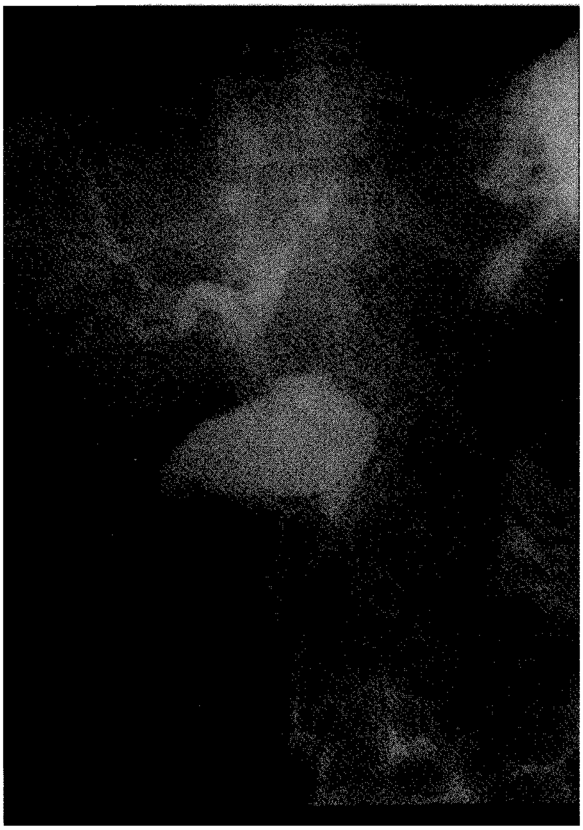

FIGURE 2 Type II choledochal cyst (choledochal diverticulum) Endoscopic Retrograde Cholangiopancreatography shows relationship of cyst to common bile duct.

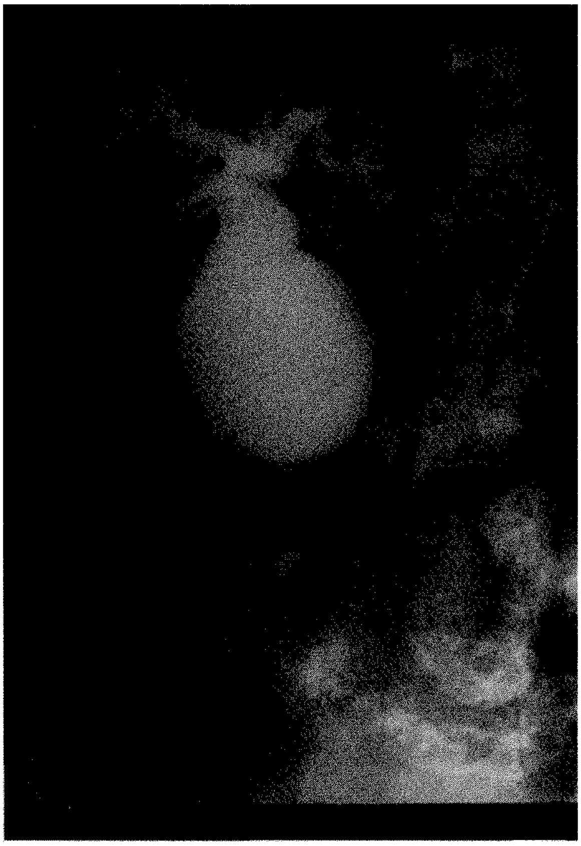

FIGURE 3 Type IB choledochal cyst. Endoscopic Retrograde Cholangiopancreatography shows focal segmental common bile duct dilatation. Note stricture of distal common bile duct (arrow).

was excised and choledochoduodenostomy was performed. The diagnosis was confirmed histologically. The patient has been followed for 29 months, and has had no complaints.

**Case 2**: A 36-yr-old female was hospitalized with jaundice, fever and RUQ pain. She had experienced intermittent RUQ pain for four years. There was no history of fever and jaundice until one week earlier. On admission her body temperature was 38.9°C. Initial laboratory data included a bilirubin of 54.4 µmol/l alkaline phosphatase of 510 U/L, g-GT of 320 U/L, leucocyte of $11.2 \times 10^9$/l. US showed dilatation of the common bile duct. This finding was confirmed by CT. ERCP demonstrated a type IB choledochal cyst with a distal common bile duct stricture (Fig. 3). Complete cyst excision, cholecystectomy and Roux-en-Y hepaticojejunostomy was performed. Two small gallstones were detected in the gallbladder at laparotomy. Histological diagnosis was choledochal cyst. The patient remained well at 22 months.

**Case 3:** A 28-yr-old female was evaluated because of intermittent epigastric pain. She had experienced the same type of pain for 5 years. There was no history of jaundice or fever. Laboratory tests unremarkable. Both US and CT demonstrated choledochal dilatation. ERCPshowed fusiform dilatation of common bile duct (type IC choledochal cyst (Fig. 4)). Complete cyst excision, Cholecystectomy and Roux-en-Y hepaticojejunostomy carried out. The pathologic evaluation was choledocal cyst. She has been perfectly well during 16 months of follow up.

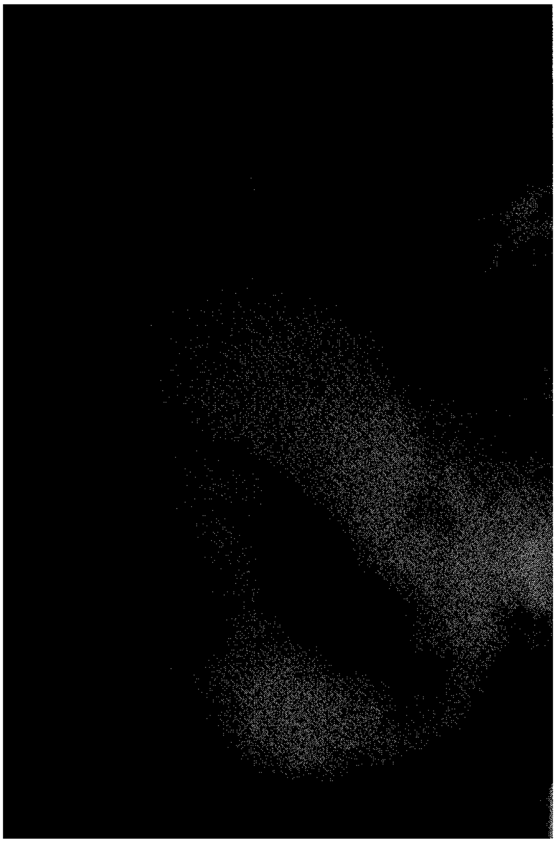

FIGURE 4 Fusiform dilatation of common bile duct (type IC) Endoscopic Retrograde Cholangipancreatography shows fusiform dilatation of common bile duct.

**Case 4:** A 29-yr-old male was admitted to the hospital because of acute pancreatitis. He reported a 1-yr history of intermittent abdominal pain with nausea. The patient had been hospitalized and acute pancreatitis was diagnosed six months before admission. Liver function tests including serum bilirubin, alkaline phoshatase, and aminotransferases were normal on admission. Serum amylase concentration was 900 U per liter. US examination of the pancreas and biliary system, and abdominal CT findings were unremarkable. Choledochocele (type III cyst) was demonstrated by ERCP (Fig. 5). He underwent cholecystectomy and choledochoduodenostomy, but the cyst could not be excised for technical reasons. Endoscopic sphincterotomy was performed later. The patient remained free from symptoms at 14 months.

**Case 5:** A 73-yr-old female was admitted to hospital because of epigastric pain, nausea and vomiting. Routine liver function test results were normal. Serum amylase concentration was 540 U per liter. The patient had undergone cholecystectomy because of cholelithiasis four years earlier. Acute pancreatitis had been diagnosed 4 months earlier at another hospital. Both US and CT revealed choledochal dilation.

ERCP demonstrated a type IV B choledochal cyst (Fig. 6). Surgery was recommended but the patient refused.

**Case 6:** A 55-yr-old female was admitted to the hospital because of fever, jaundice and abdominal pain. She was well 3 months earlier, when she began to have severe RUQ pain and nausea. 20 days before admission she had experienced fever and jaundice. On admission her temperature was 39°C, the pulse was 104, blood pressure was 90/60 mm Hg. Initial laboratory findings were as follows: hemotocrit 0.267 leucocyte $34\times10^9/l$ with a left shift, platelet $188\times10^9/l$, total bilirubin 122.4 μmol/l, direct bilirubin 91.8 μmol/l, alkaline phosphatase 1370 U/L, γ-GT 615 U/L, AST 168 U/L, ALT 195 U/L. CT revelad multiple abscesses in the liver. At laparotomy, the gallbladder was found to be perforated and there were many intrahepatic biliary cysts which contained multiple bile stones and abscesses. Cholecystectomy, abscesses drainage and choledochotomy were carried out. ERCP findings which was performed after laparotomy were consistent with Caroli's disease and bile stones were demonstrated both in the cysts and in the common bile duct (Fig. 7). Histological examination of the gallbladder revealed adenocarcinoma. In addition, the diagnosis of intrahepatic biliary cyst was confirmed histologically.

### DISCUSSION

It is well known that Choledochal cysts are uncommon biliary tract abnormalities. Between January 1991 and December 1994, six choledochal cysts were diagnosed out of 326ERCP procedures at Çukurova University School of Medicine

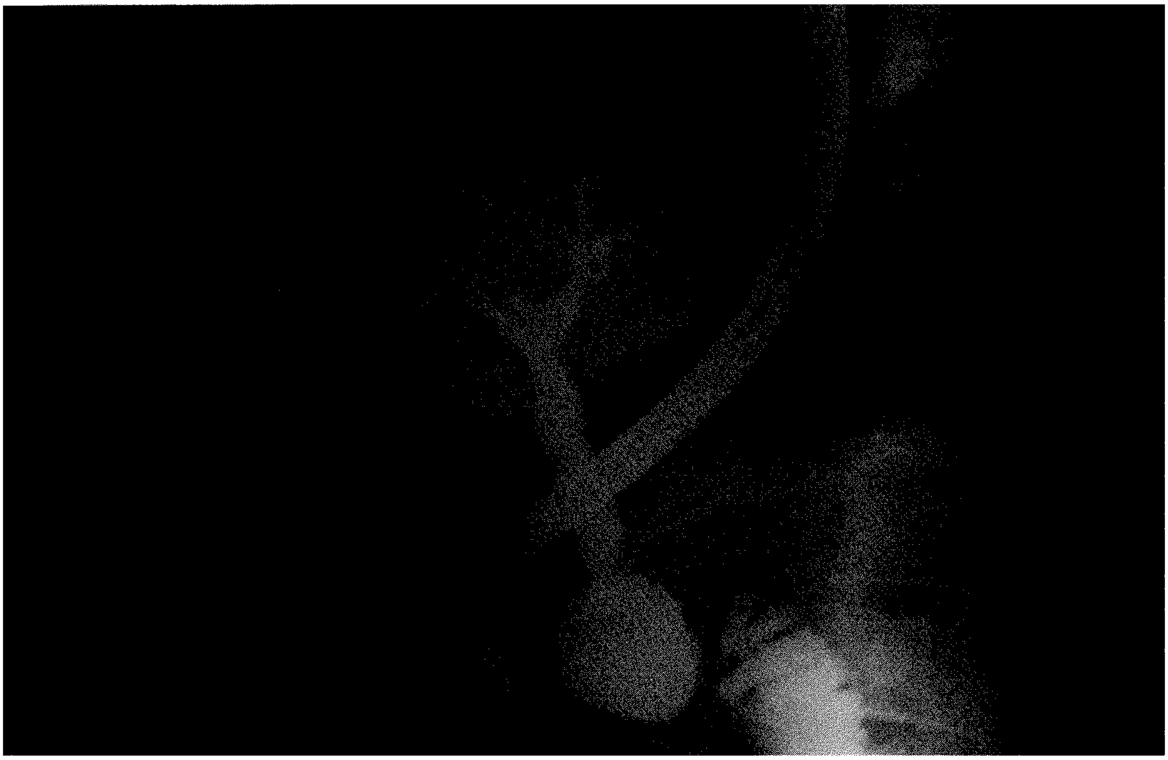

FIGURE 5 Endoscopic Retrograde Cholangiopancreatography demonstrates focal dilatation of intraduodenal portion of common bile duct (choledochocele, type III).

Adana, Turkey. Choledochal cysts occur three of four times more common in females than males (1,2,5). The ages of the reported cases range from neonates to 87 years (1, 5). However 60% of cases are diagnosed before the age of 10 (1). five out of six of our cases were female and all of them were diagnosed in adult life. The youngest patient in our series was a 28-year-old female and the eldest one was a 73-year old female.

The classic clinical triad for choledochal cysts are abdominal pain, abdominal mass and jaundice which is found in only a third of cases (range 13–68%) (7, 10). This triad was not observed in our patients. The most frequent symptom was abdominal pain in our series. In the literature symptoms were generally chronic also intermittent and duration of symptoms varies from one week to more than 40 years (1). In our patients symptoms of choledochal cysts are most often related to their complication especially to biliary obstruction and stasis (1). Many complications such as cholelithiasis, choledocholithiasis, primary cysts stones, cholangitis, pancreatitis, secondary biliary cirrhosis, intrahepatic abscess and biliary cancer have been reported (7,13,21, 24–30) > Cholangitis and pancreatitis are frequently observed in bile duct cysts (14,29). Biliary cirrhosis develops in proportion to duration and degree of obstruction (7). Primary cyst stones occur in 8% of patients (11,29). Associated hepatobiliary diseases were detected in five of six patients in our series, including cholelithiasis in three, common bile duct structure in one, acute pancreatitis in two, intrahepatic abscess and intrahepatic cyst stones in one. The incidence of hepatobiliary diseases in our patients were similar to previously reported cases in the literature (14,29). Both intrahepatic abscess and intrahepatic cysts stones were observed in our patient with Caroli's desease.

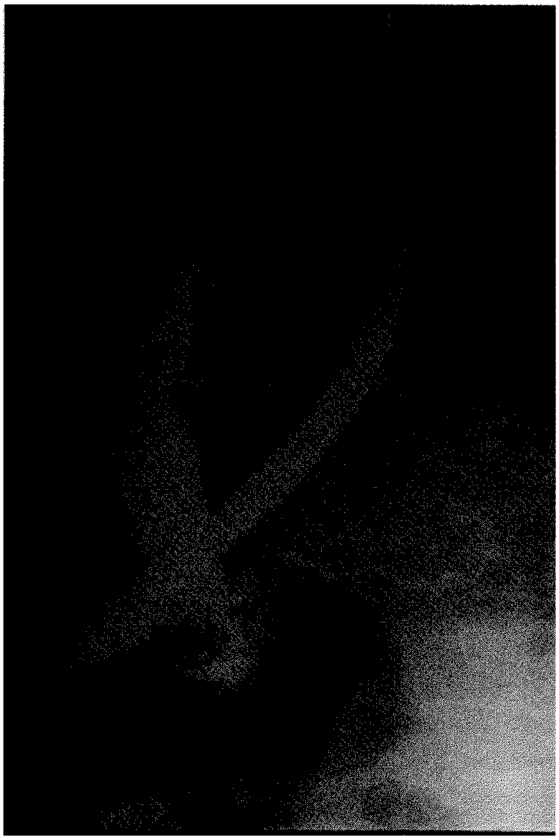

FIGURE 6 Endoscopic Retrograde Cholangiopancreatography shows extrahepatic cysts (type IV B).

The reported incidence of biliary cancer related to choledochal cysts ranges between 2, 5% and 28% (1, 20). Carcinogenesis in choledochal cysts still remains controversial. However, some bile acid fraction (lithocholic and deoxycholic acids), and regurgitated pancreatic enzymes in bile (activated tripsin, elastase I, and phospholipase A2) are hazardous to the epithelium, and may promote carcinoma under conditions of infection, inflammation, bile stasis, decreased trypsin inhibitors, and the presence of enterokinase (30,31). Carcinoma generally develops in the extrahepatic bile duct (26,32). Adenocarcinoma is the most common histology, squamous and undifferentiated carcinomas are also reported, but less frequently (24,31,33). Only one of our patients had gallbladder carcinoma. Although in the literature most of the biliary cancers are developed in patients with type I choledochal cysts, our patient with gallbladder carcinoma had Caroli's disease.

In our experience ERCP provides detailed analysis of the anatomy of the biliary tree and pancreaticobiliary junction. It is generally agreed that in cases of choledochal cyst, the delination of the anatomy of the biliary tree by cholangiopancreatography is useful for planning appropriate treatment (14,18). Both ERCP and percutaneus transhepatic cholangiography have been used in adults when the jaundice is mild and the clinical signs minimal (15,18). Analysis of ERCP studies in adults has shown abnormalities of the pancreatobiliary junction, which have provided clues to the etiology of the choledochal cysts (2,8,9). Few report in the English language literaturehave described the use of ERCP in children who have choledochal cysts (15,17,18). Technical difficulty, the need for general anesthesia, and complications such as cholangitis have limited the use of this investigative method in children (18). For the infant or child, generally ultrasound and/or radionuclide scanning are adequate when combined with intraoperative cholangiography (18).

It is accepted that management of the choledochal cysts is surgical although the type of surgery has beeen contriversial (1,2,14,20,23,34–37). Complete cyst excision and Roux-en-Y hepaticojejunostomy are recommended as the treatment of choice for adults to prevent pancreatitis and possibly to reduce the risk of malignancy (1, 5,20, 36). The disadvantages of cyst excision are prolonged operating time and the risk of injury to the hepatic artery, portal vein or pancreatic duct (13). Long-term complications of choledochal cyst surgery include stone formation within the intrahepatic bile ducts or within the intrapancreatic bile ducts (37). Lesser procedures such as cystoduodenostomy have been associated with high morbidity rates and with the potential for malignant change in the biliary tree (23). In selected cases choledochocele may be effectively managed by endoscopic sphincterotomy (14).

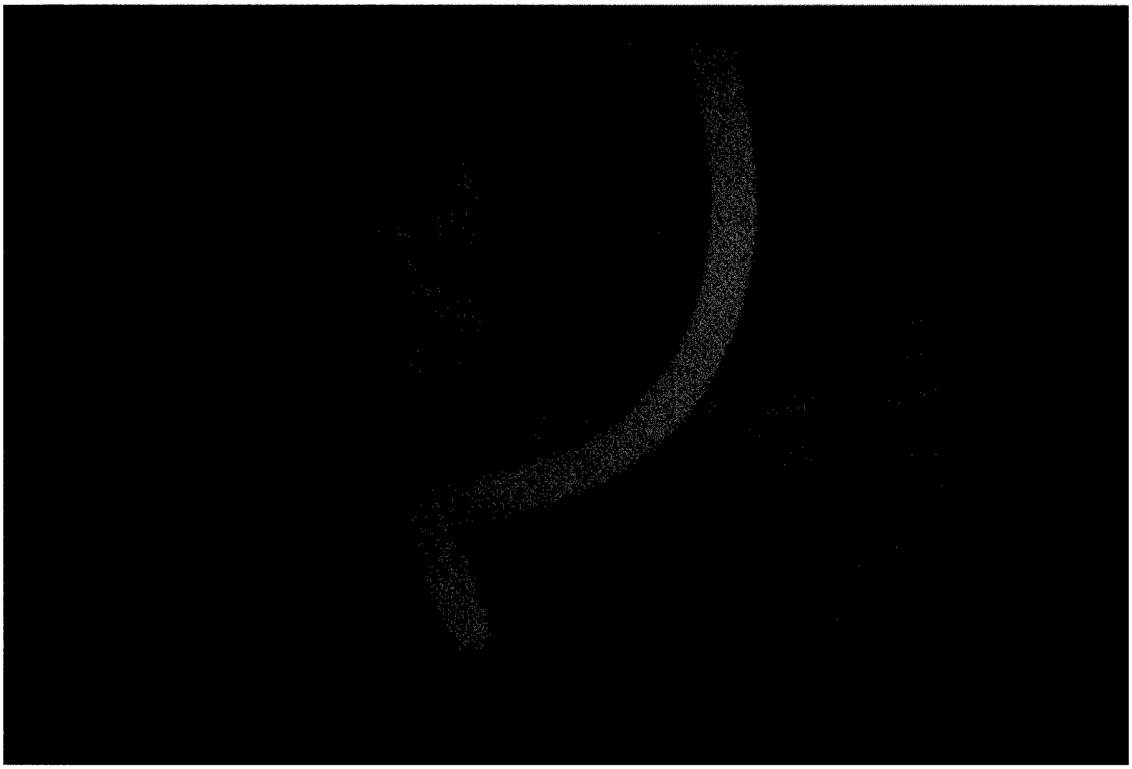

FIGURE 7 Endoscopic Retrograde Cholangiopancreatography demonstrates multiple intrahepatic cysts (Caroli's disease type V). Note bile stone in the intrahepatic cyst (short arrow) and in distal common bile duct (long arrow).

Unilateral Caroli's disease may be treated by hepatic resection (13). In 2 of our cases complete cyst excision and Roux-en-Y he paticojej unostomy were performed (Cases 2,3). One patient (Case 1) underwent total cyst excision and choledochoduodenostomy. In case 4 (type III choledochal cysts) cholecystectomy and choledochoduoedenostomy were performed at laparotomy but the cyst could not be excised. Endoscopic sphincterotomy was carried out later in this patient. In case 6 (Caroli's disease) cholecystectomy, abscess drainage and choledochotomy were the surgical procedures.

In conclusion although our population does not represent all Turkey our results indicate that this anomaly of the biliary system should be considered more seriously in the Turkish population. We also concluded that ERCP has a high positive diagnostic rate and it not only delineated the choledochal cyst, but also showed additional anomalies in the biliary tree.

### *References*

[1] Crittenden, S.L. and Mc Kinley, M.J. (1985). Choledochal cyst-clinical features and classification, *Am J Gastroenterol*, **80**, 643–47.

[2] Suchy, F.J. (1993). Disorders of the biliary tract in infancy and childhood. In: Sleisenger MH, Fortran Js, eds. Gastrointestinal disease. Pathophysiology/ Diagnosis/Management/Philadelphia: WB Saunders. 1747–64.

[3] O'Neill, J.A., Templeton, J.M. and Sechnaufer, L., *et al.* (1987). Recent experience with choledochal cyst, *Ann Surg.*, **205**, 533–40.

[4] Tan, K.C. and Howard, E.R. (1988). Choledochal cyst: a fourteen-year surgical experience with 36 patients, *Br J Surg.*, **75**, 892–95.

[5] Dewilde, V.G., Elewaut, A.G. and De Vas, M.P., *et al.* (1991). Choledochal cysts in the adult. *Endoscopy*, **23**, 4–7.

[6] Savader, S.J., Benti, J.F. and Venbrux, A.C., *et al.* (1991). Choledochal cysts: Classification and cholangiographic appearence, *AJR.*, **156**, 327–31.

[7] Alonso-lej, F., Rever, W.B. and Pessagno, D.J. (1959). Congenital choledochal cyst with a repot of two, and analysis of 94 cases, *Int Abstr Surg.*, **108**, 1–30.

[8] Babbitt, D.P. (1969). Congenital Choledochal cysts: New etiological concept based on anomalous relationships of the common bile duct and pancreatic bulb, *Ann Radiol*, **12**, 231–40.

[9] Davenport, M., Stringer, M.D. and Howard, E.R.

(1995). Biliary Amylase and Congenital Choledochal Dilatation, *J Pediatr Surg.*, **30**, 474–47.
[10] Todani, T., Watanabe, Y. and Narusue, M, *et al.* (1977). Congenital bile duct cyst. Classification, operative procedures, and review of 37 cases including cancer arising from choledochal cyst, *J. Am.*, **134**, 263–9.
[11] Yamaguchi, M. (1980). Cogenital choledochal cyst. Analysis of 1,433 patients in the Japanese literature, *Am J Surg.*, **140**, 653–7.
[12] Klein, G.M. and Frost, S.S. (1981). Newer imaging modalities for the preoperative diagnosis of choledochal cyst, *Am J Gastroenterol*, **76**, 148–52.
[13] Nume Hoyo, M., Lees, C.D., and Hermann, R.E. (1982). Bile duct cysts, *Am J Surg.*, **144**, 295–9.
[14] Venu, R.P., Geenen, W.J. and Hogan, W.J, *et al.* (1984). Role of endoscopic RetrogradeCholangiopancreatography in the diagnosis and treatment of choledochocele, *Gastroenterology*, **87**, 1144–9.
[15] Wiedmeyer, D.A., Stewart, E.T. and Dodds, W.J., *et al.* (1989). Choledochal cyst. Findings on cholangiopancreatography with emphasis on ectasia of the common channel, *AJR*, **153**, 969–72.
[16] Thatcher, B.S., Sivak, M.V. and Hermann, R.E., *et al.* (1986). ERCP in evaluation and diagnosis of choledochal cyst: report of five cases, *Gastrointest Endosc.*, **32**, 27–31.
[17] Urakami Y, Seki H. and Kiski S, (1977). Endoscopic Retrogade Cholangiopancreatography performed in children, *Endoscopy*, **9**, 86–91.
[18] Sharma, A.K., Wakhlu, A. and Sharma, S.S. (1995). The role of Endoscopic RetrogadeCholangiopancreatography in the Management of Choledochal Cysts in Children, *J Pediatr Surg.*, **30**, 65–67.
[19] Todani, T., Watanabe, Y. and Mizuguchi, T., *et al.* (1981). Hepaticoduodenostomy at the hepatic hilum after excision of choledochal cyst, *Am J Surg.*, **142**, 584–7.
[20] Nagorney, D.M., McIlrath, D.C. and Adson, Am. (1984). Choledochal cysts in adults : Clinical management, *Surgery*, **96**, 656–63.
[21] Oldham, K.T., Hart, M.J. and White, T.T. (1981). Choledochal cysts presenting in late childhood and adulthood, *Am J Surg.*, **141**, 568–71.
[22] Scudamore, C.H., Hemming, A.W. and Teare, J.P., *et al.* (1994). Surgical Management of Choledochal Cysts, *Am J Surg.*, **167**, 497–500.
[23] Todani, T., Watanabe, Y. and Urishihara, N., *et al.* (1995). Bilary Complications After Excisional precedure for Choled Cyst, *J Pediatr Surg.*, **30**, 478–81.
[24] Todani, T. and Tabuchik Watanabe, Y., *et al.* (1979). Carcinoma arising in the wall of congenital bile duct cyst, *Cancer*, **44**, 1134–41.
[25] Sameshima, Y., Uchimura, M. and Muto, Y., *et al.* (1987). Coexistent carcinoma in congenital dilatation of the bile duct and anomalous arrangement of the pancreaticobile duct. Carcinogenesis of coexistent gall bladder carcinoma, *Cancer*, **60**, 1883–90.
[26] Todani, T., Watanabe, T. and Urushira, N., *et al.* (1994). Choledochal cysts, pancreatobiliary malunion, and cancer, *J Hep Bil Pancr Surg.*, 247–51.
[27] Voyles, C.R., Smadja, C. and Shands, C., *et al.* (1983). Carcinoma in choledochal cysts. Ageraleted incidence, *Arch Surg.*, **118**, 986–8.
[28] Ozawa, K., Yamada, T. and Matumoto, Y., *et al.* (1980). Carcinoma arising in a choledochocele, *Cancer*, **45**, 195–97.
[29] Matsumoto, Y., Uchida, K. and Nakase, A., *et al.* (1977). Congenital cystic dilatation of the common bile duct as a cause of primary bile duct stone, *Am J Surg.*, **134**, 346–52.
[30] Swisher, S.G., Cates, J.A. and Hunt, K.K., *et al.* (1994). Pancreatitis Associated with Adult choledochal cysts, *Pancreas*, **9**, 633–37.
[31] Ono, J., Sakoda, K., Akita, H. and Akita, H., (1982). Surgical aspect of cystic dilatation of the bile duct. An anomalous junction of the pancreaticobiliary tract in adults, *Ann Surg.*, **195**, 203–8.
[32] Funabiki, T., Sugive, K. and Matsubra, T., *et al.* (1990). Bile acides and biliary carcinoma in pancreaticobiliary maljunction, *Keio J Med.*, **40**, 118–22.
[33] Kato, T., Matsuda, K. and Kayaba, H., *et al.* (1989). Pathology of anomalous junction of the biliary tract and unclear atypia of the biliary epithelium, *Keio J Med.*, **38**, 167–76.
[34] Powell, C.S., Sawyers, J.L. and Reynolds, V.H. (1981). Management of adult choledochal cysts, *Ann Surg.*, **193**, 666–7.
[35] Lilly, J.R. (1979). The surgical treatment of choledochal cyst, *Surg Gynecol obstet.*, **149**, 36–42.
[36] Schier, F., Clausen, M. and Kouki, M., *et al.* (1994). Late Restlts in the Management of Choledochal Cysts, *Eur J Pediatr Surg.*, **4**, 141–44.
[37] Miyano, T., Yamataka, A. and Kato, Y., *et al.* (1995). Cholodochal Cysts: Special Emphasis on the Usefulness of Intraoperative Endoscopy, *J Pediatr Surg.*, **30**, 482–84.

### COMMENTARY

This report 6 adult patients with choledochal cysts nicely illustrates the diversity of clinical presentations, the anatomic variations typical of the disorder, and several important established management principles. It also underscores the differences between adult and pediatric patients

Cases 1, 2 and 3 illustrate the principle that total transmural excision of the cyst is preferred when possible and that reconstruction is best done with a Roux-en-Y hepaticojejunostomy. Late diagnosis, incomplete excision, or internal drainage without excision lead predictably to serious problems such as acute pancreatitis, complex biliary stone disease and adenocarcinoma of the extrahepatic biliary tract.

Children are perhaps a more consistent group of patients in that the diagnosis can be made predictably using prenatal maternal ultrasound screening or a combination of biliary scintigraphy and ultrasound. In most reports, children represent the majority of these patients and they can generally be recognized and treated without the need for morbidity of preoperative endoscopic retrograde cholangiopancreatography (ERCP). In contrast, adults present with more variable symptoms and a more complex differential diagnosis, so it appears that (ERCP) has a greater role in the latter group.

In summary, this contribution emphasizes the need for seasoned surgical judgement and the incorporation of contemporary endoscopic techniques in the management of older patients with choledochal cysts.

Dr. K.T. Oldham
Duke University of Medical Centre
Department of Surgery
P.O. Box 3815
Durham NC 27710
USA

